# Preoperative and intraoperative ultrasound aids removal of migrating plant material causing iliopsoas myositis via ventral midline laparotomy: a study of 22 dogs

**DOI:** 10.1186/s13028-017-0280-5

**Published:** 2017-02-14

**Authors:** Francesco Birettoni, Domenico Caivano, Mark Rishniw, Giulia Moretti, Francesco Porciello, Maria Elena Giorgi, Alberto Crovace, Erika Bianchini, Antonello Bufalari

**Affiliations:** 1 Department of Veterinary Medicine, University of Perugia, Via San Costanzo 4, 06126 Perugia, Italy; 2 Department of Clinical Sciences, Cornell University, Ithaca, NY 14853 USA; 3 Veterinary Information Network, Davis, CA 95616 USA

**Keywords:** Intraoperative ultrasound, Grass awn, Myositis, Canine

## Abstract

**Background:**

Migrating plant material is often suspected clinically to be the underlying cause of iliopsoas myositis in the dog, but cannot always be found pre- or intraoperatively. In most cases, recurrence of clinical signs is related to failure to remove the plant material. Preoperative ultrasonography can be useful to visualize migrating plant material and to determine anatomical landmarks that can assist in planning a surgical approach. The purpose of the present study was to report the role of intraoperative (intra-abdominal) ultrasonography for visualizing and removing the plant material from iliopsoas abscesses using a ventral midline laparotomy approach.

**Results:**

A retrospective case series of 22 dogs with iliopsoas muscle abnormalities and suspected plant material was reported. Preoperative visualization and subsequent retrieval of the plant material was performed during a single hospitalization. In all 22 dogs, the plant material (including complete grass awns, grass awn fragments and a bramble twig) was successfully removed via ventral midline laparotomy in which intraoperative ultrasonography was used to direct the grasping forceps tips to the foreign body and guide its removal. In 11 of these 22 dogs, the plant material was not completely removed during prior surgery performed by the referring veterinarians without pre- or intraoperative ultrasonography. Clinical signs resolved in all dogs and all dogs resumed normal activity after successful surgical removal of the plant material.

**Conclusion:**

Intraoperative ultrasonography is a safe and readily available tool that improves success of surgical removal of plant material within the iliopsoas abscesses via ventral midline laparotomy. Moreover, ultrasonographic findings of unusual plant material can be useful in planning and guiding surgical removal, by providing information about the size and shape of the foreign body.

## Background

Iliopsoas muscle disease has been well described in canine patients. This pathological process includes traumatic injury [1–3], muscle strain injury [4], primary haemangiosarcoma [5], fibrotic myopathy [6–8] and abscessation [9, 10]. With iliopsoas abscessation, pus accumulates within and around the iliopsoas muscles, often producing a draining cutaneous fistula just cranial to the ileum [10]. Most iliopsoas abscesses result from plant material, such as migrating grass awns, and various diagnostic techniques, including radiology, contrast radiology, ultrasonography, computed tomography (CT), magnetic resonance imaging (MRI), have been used to investigate fistulae associated with iliopsoas abscesses [10–19]. Additionally, these imaging tools help determine anatomical landmarks that can be used in planning a surgical approach. Ideally, treatment of iliopsoas myositis secondary to migrating plant material requires removal of the foreign body, coupled with antibiotic therapy. Plant material is often suspected clinically to be the underlying cause, but cannot always be found because it can be difficult to identify during an open surgery or it has migrated out of the fistula [11, 13, 18, 19].

To the author’s knowledge, no study in the veterinary literature has documented the role of intraoperative ultrasonography for visualizing and removing plant material from iliopsoas abscesses using a ventral midline laparotomy. Therefore, we sought to report a method to routinely investigate and treat dogs with iliopsoas myositis due to migrating plant material.

## Methods

Electronic medical records of dogs evaluated at the Veterinary Teaching Hospital of Perugia University between January 2012 and October 2015 were searched to identify those in which ultrasonographic findings of the iliopsoas muscle region were compatible with plant material migration (identification of iliopsoas abscessation/myositis with hyperechoic structures of variable length consistent with a foreign body that cast characteristic shadows through the ultrasonographic image). Dogs with history of trauma to the abdominal cavity were excluded from the study. All dogs required a recorded follow-up >6 months after ultrasonographic exam and surgery. For all cases that satisfied these criteria we reviewed history, signalment, clinical, radiological and CT findings (if available), as well as surgical findings, other treatments, and outcome.

Ultrasonographic examination of the iliopsoas muscle region was performed on awake dogs positioned in left and right lateral recumbency with an ultrasound system^1^ equipped with a 5- to 8-MHz microconvex transducer (see footnote 1) after clipping fur over the abdomen and both flanks. Left and right iliopsoas muscle regions were scanned in a cranial to caudal direction with the scan plane held parallel and then perpendicular to the spine. Where possible, the plant material was identified, based on specific, previously described, imaging criteria [15, 17, 20–22]. Subsequently, adjacent anatomical landmarks (e.g., kidneys, aorta, caudal vena cava, renal arteries and their distance from the suspected foreign body) were identified to help the surgeon during intra-abdominal exploration.

In 2 cases, ultrasonography was performed with the dog positioned in dorsal recumbency under deep sedation because these dogs displayed profound abdominal guarding when unsedated (hunched over, unwilling to relax and have the hindlimbs retracted caudally to extend the abdominal musculature), and did not allow an adequate and systematic scan of the sublumbar region and in particular of iliopsoas muscles.

All dogs were hospitalized for 1–2 days after preoperative ultrasonographic visualization of the suspected migrating plant material while awaiting surgical exploration.

Intraoperative (intra-abdominal) ultrasonography was performed by use of a microconvex probe (see footnote 1) encased in a sterile protective cover^2^ and positioned directly on the affected region of the iliopsoas muscles (identified by preoperative anatomical landmarks and intraoperative visual inspection) to precisely localize the suspected plant material and guide complete removal.

Anaesthetic and analgesic protocols were determined on an individual basis by the attending anaesthetist. All dogs were positioned in dorsal recumbency for the procedure and a ventral midline laparotomy was performed through a 13–22 cm incision, depending on the size of the dog. The surgical field was isolated with laparotomy gauze and abdominal muscles retracted by a Balfour self-retaining retractor to allow the intraoperative ultrasonographic exploration of the iliopsoas muscles. Once the plant material was identified, an 18 or 20 G spinal needle was introduced through the iliopsoas muscle using ultrasonographic guidance and the bevel was positioned close to the foreign body to act as guide for a #10 scalpel blade. The surgeon then made a small incision (1–2 cm) through the ventral iliopsoas epimysium under ultrasonographic guidance, and introduced grasping forceps through this incision to retrieve the plant material. When a grass awn was identified, the surgeon carefully grasped the tip of the grass awn and gently extracted it through the incision. Because of the harpoon-like shape of grass awns, care was taken not to grasp the grass awn by the barbs, so as to avoid unintentional fragmentation. Various types of grasping forceps were used for the plant material removal, depending on the size of the patient and the surgeon’s preference: 7–9 cm Hartmann alligator forceps, 8 cm Kelly curved haemostatic forceps, or 24 cm Kantrowitz thoracic clamps. After plant material removal, intraoperative ultrasonography of the affected region was again performed to confirm its complete removal. The muscle incision was lavaged with sterile saline solution and aspirated, then the incision was sutured with single absorbable sutures. When a minimum amount of fluid was recovered, this approach provided an adequate debridement of the affected region. Omentalization of the abscess cavity was performed in dogs with large amounts of fluid and larger abscess cavities. No cases required extensive debridement or the insertion of a drainage tubes. Postoperative treatment consisted of daily wound and fistula (if present) care, pain management [23] and antibiotic therapy. Cefaxolin (30 mg/kg q12 h) in association with enrofloxacin (5 mg/kg q24 h) were intravenously administrated before the induction and continued in the following days. Antibiotics were changed on the basis of results of microbial culture and susceptibility testing of both the samples collected from the affected region and any retrieved plant material.

Short-term outcome was assessed by reviewing recheck examination records (usually 10 days and 1 months after the surgery), and long-term outcome was reviewed by telephone consultation with the owners or the referring veterinarians. Descriptive data are reported. Statistical analyses were not performed.

## Results

Twenty-two dogs with a diagnosis of suspected migrating plant material in the iliopsoas muscle region met the study inclusion criteria. Breeds included English Setter (n = 8), Springer Spaniel (3), Italian Bloodhound (3), Kurzhaar (3), German Shorthaired Pointer (1), Epagneul Breton (1), English Pointer (1), and mixed (3). Of the 22 dogs, 9 were female and 13 were male, with a median age of 4.3 years (range, 1–10 years) and median weight of 19.2 kg (range, 7.5–40 kg). All dogs had been treated with antimicrobial agents and 11 dogs had undergone one to two (n = 4) surgical explorations (via lateral or ventral midline laparotomy) prior to initial evaluation at the Veterinary Teaching Hospital.

In 12/22 dogs, historical findings we considered related to the migration of plant material through the airways towards the iliopsoas muscles, included pyrexia, cough and dyspnea in the previous spring/summer season.

Relevant clinical signs included flank swelling and pain (n = 20), pyrexia (n = 16), depression (n = 16), hindlimb lameness (n = 11) and anorexia (n = 10). Eight dogs had cutaneous fistulae in the dorsal midlumbar region.

**Fig. 1:**
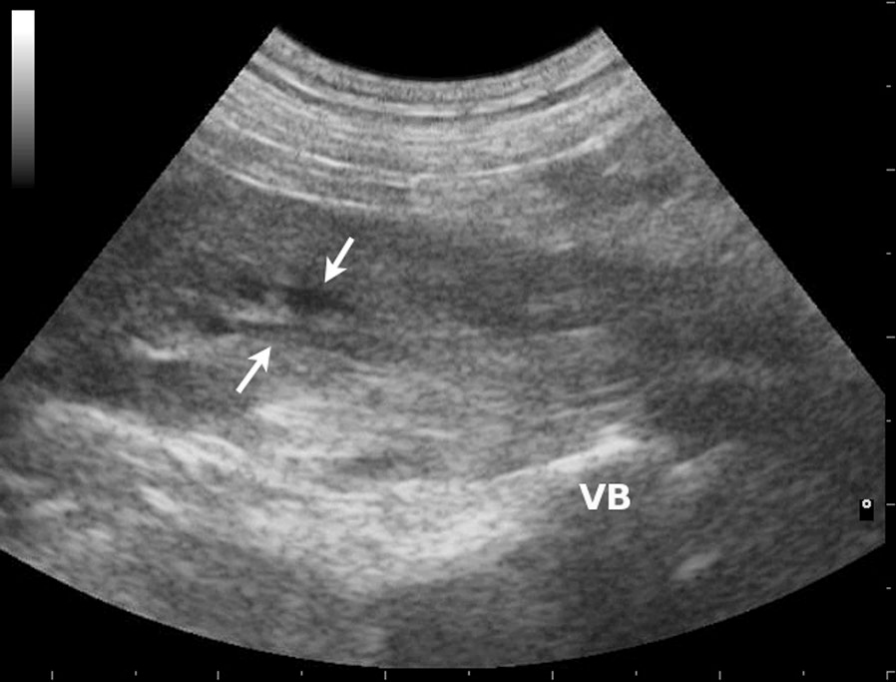
Ultrasonographic image (longitudinal plane) of an affected iliopsoas muscle. Loss of typical fascicular architecture with inhomogeneously hypoechoic appearance and accumulation of flocculent fluid (*arrows*) is present. *VB* vertebral body

**Fig. 2:**
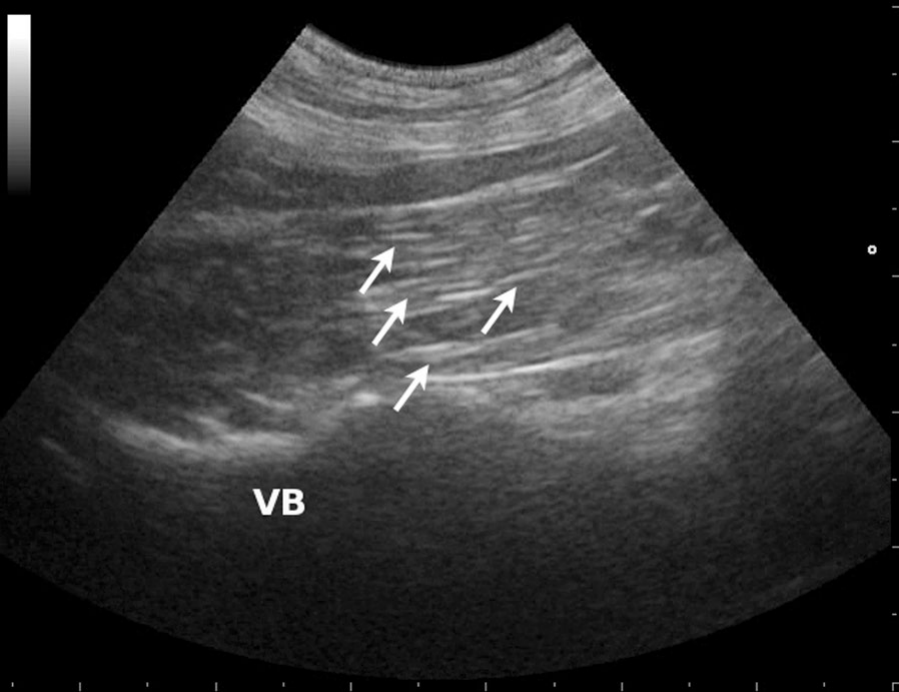
Ultrasonographic image (longitudinal plane) of a normal iliopsoas muscle. The muscle is homogeneously echogenic with low echo intensity linear appearance (*arrows*) because of reflections of perimysial connective tissue. *VB* vertebral body

Iliopsoas muscle abnormalities were detected within the left (n = 14), right (n = 6) or both (n = 2) iliopsoas muscles with ultrasonographic imaging. In all unilaterally affected dogs the affected muscle appeared swollen with a loss of typical fascicular architecture and in homogeneously hypoechoic with accumulation of flocculent fluid (Fig. 1). A mild to moderate amount of fluid was also present in the subcutaneous tissue in the 8 dogs with a fistula. In 20 dogs, the tissues surrounding the muscle capsule (epimysium) appeared moderately hyperechoic. The normal (contralateral) iliopsoas muscle was homogeneously echogenic with low echo intensity and a speckled appearance (in the transverse plane) or linear/pinnate appearance (in the longitudinal plane) because of reflections of perimysial connective tissue (Fig. 2). The suspected plant material, surrounded by a focal hypoechoic zone (consistent with myositis) was preoperatively visualized in all 22 dogs. Plant material was visualized within the affected iliopsoas in dogs with unilateral abnormalities; in the 2 dogs where both iliopsoas muscles showed ultrasonographic abnormalities, the grass awn was observed within the affected iliopsoas (the right iliopsoas in both instances) and within in the medial portion of the left iliopsoas muscle, essentially straddling the midline.

**Fig. 3:**
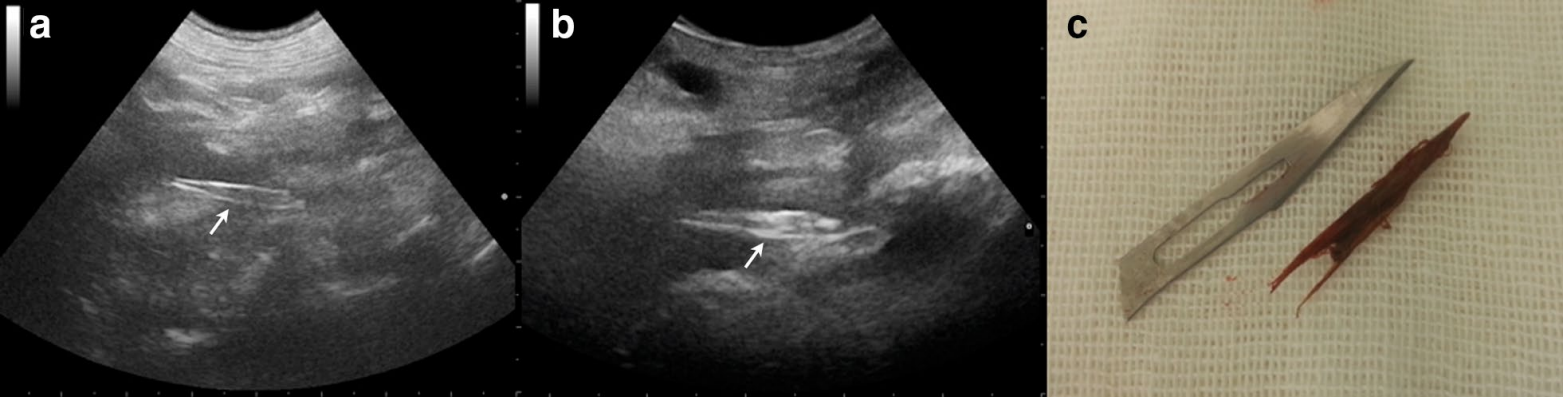
Ultrasonographic images (**a**, **b**) and intraoperative photograph (**c**) of a 3–year-old German Shorthaired Pointer with a grass awn in the left iliopsoas muscle. **a** Transabdominal ultrasonographic image of a spindle-shaped hyperechoic foreign body consistent with a grass awn (*arrow*). **b** Intraoperative ultrasonographic image of the same awn shown in* panel A* (*arrow*). **c** Photograph of the grass awn after the removal

**Fig. 4:**
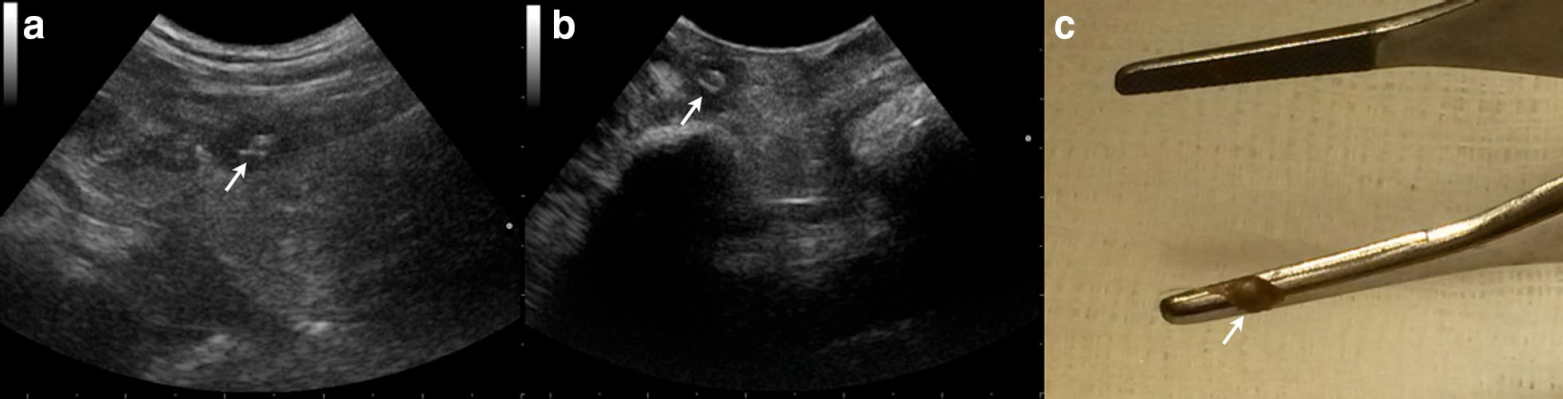
Ultrasonographic images (**a**, **b**) of a 3–year-old English Setter with an oval plant material in the left iliopsoas muscle and intraoperative photograph of the awn after removal (**c**). **a** Transabdominal ultrasonographic image showing the 0.3-cm-long awn (*arrow*) with a small linear hyperechoic structure compatible with a barb. The foreign body is surrounded by a hypoechoic zone consistent with myositis. **b** Intraoperative ultrasonographic image of the plant material in ** a** confirming its presence in the left iliopsoas muscle. **c** The plant material is shown

**Fig. 5:**
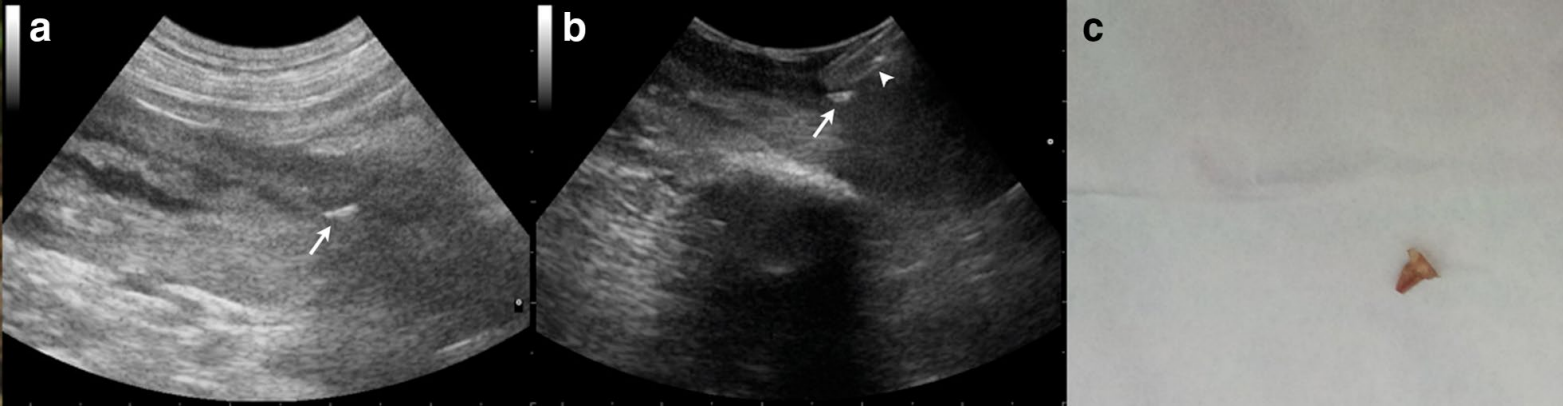
Ultrasonographic images (**a**, **b**) of a 3–year-old English Setter with a fragment of plant material and intraoperative photograph of the fragment after removal (**c**). **a** Transabdominal ultrasonographic image of a small, linear and hyperechoic foreign body (*arrow*) in the left iliopsoas muscle. **b** Intraoperative ultrasonographic image of the foreign body (*arrow*) in* panel A* confirming its presence. The arm of a Hartmann forceps (*arrowhead*) is visible in proximity to the foreign body. **c** Photograph of the fragment after removal

**Fig. 6:**
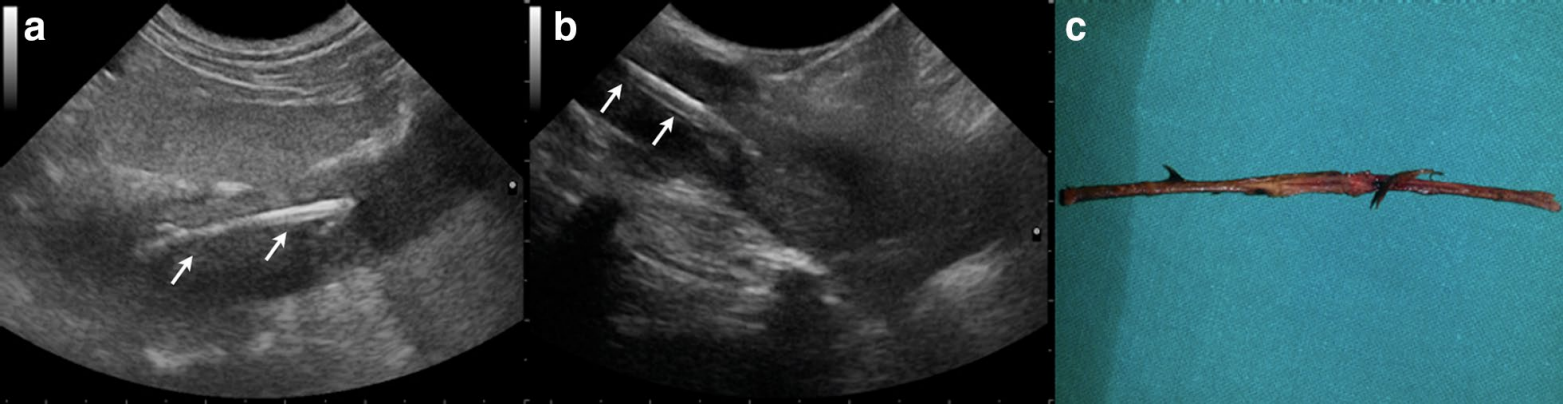
Ultrasonographic image (**a**, **b**) of a 5–year-old Kurzhaar with a migrating plant material in the right iliopsoas muscle and intraoperative photograph of the foreign body after removal (**c**). **a** Transabdominal ultrasonographic image of a 3.5 cm, linear, shadowing and high hyperechoic foreign body (*arrows*). **b** Intraoperative ultrasonographic image of the foreign body (*arrows*) in* panel A* confirming its presence in the iliopsoas muscle. **c** Photograph of the bramble branch after removal

Grass awns typically appeared ultrasonographically as spindle-shaped, shadow casting, hyperechoic structures of variable length (range, 0.5–2.5 cm) (Fig. 3). In 1 dog, the plant material appeared as a 0.3 cm, oval-shaped structure with a small linear hyperechoic projection compatible with a barb (Fig. 4). In 2 dogs that had undergone prior attempts at grass awn removal by the referring veterinarian, fragments of plant material were visualized by preoperative ultrasonography (Fig. 5). In one dog, a 3.5 cm, linear, shadowing, highly hyperechoic foreign body (ultimately identified as a bramble branch) was visualized (Fig. 6).

**Fig. 7:**
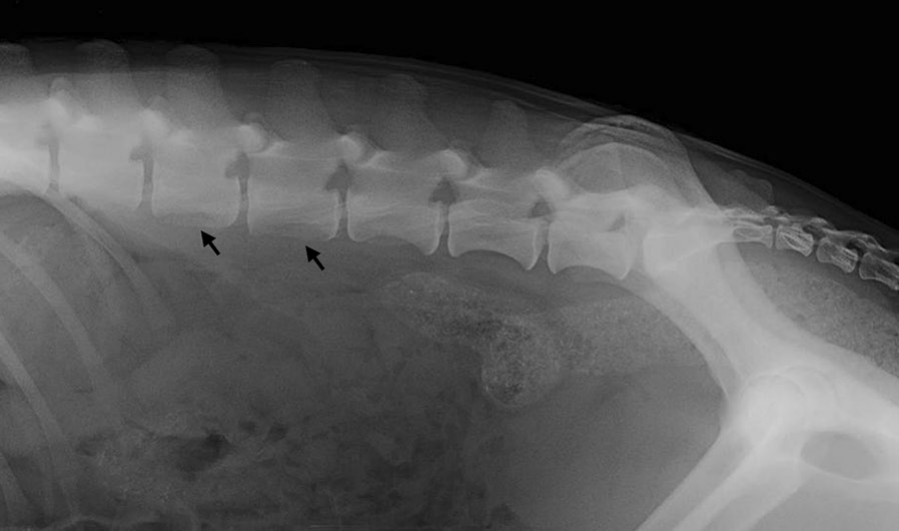
Lateral projection of the lumbosacral spine of a dog with a foreign body in the iliopsoas muscles; the radiograph shows bone proliferations of the ventral part of the vertebral body of L4 and L3 (*black arrows*), and an irregularity of the periosteum of the ventral profile of L2

**Fig. 8:**
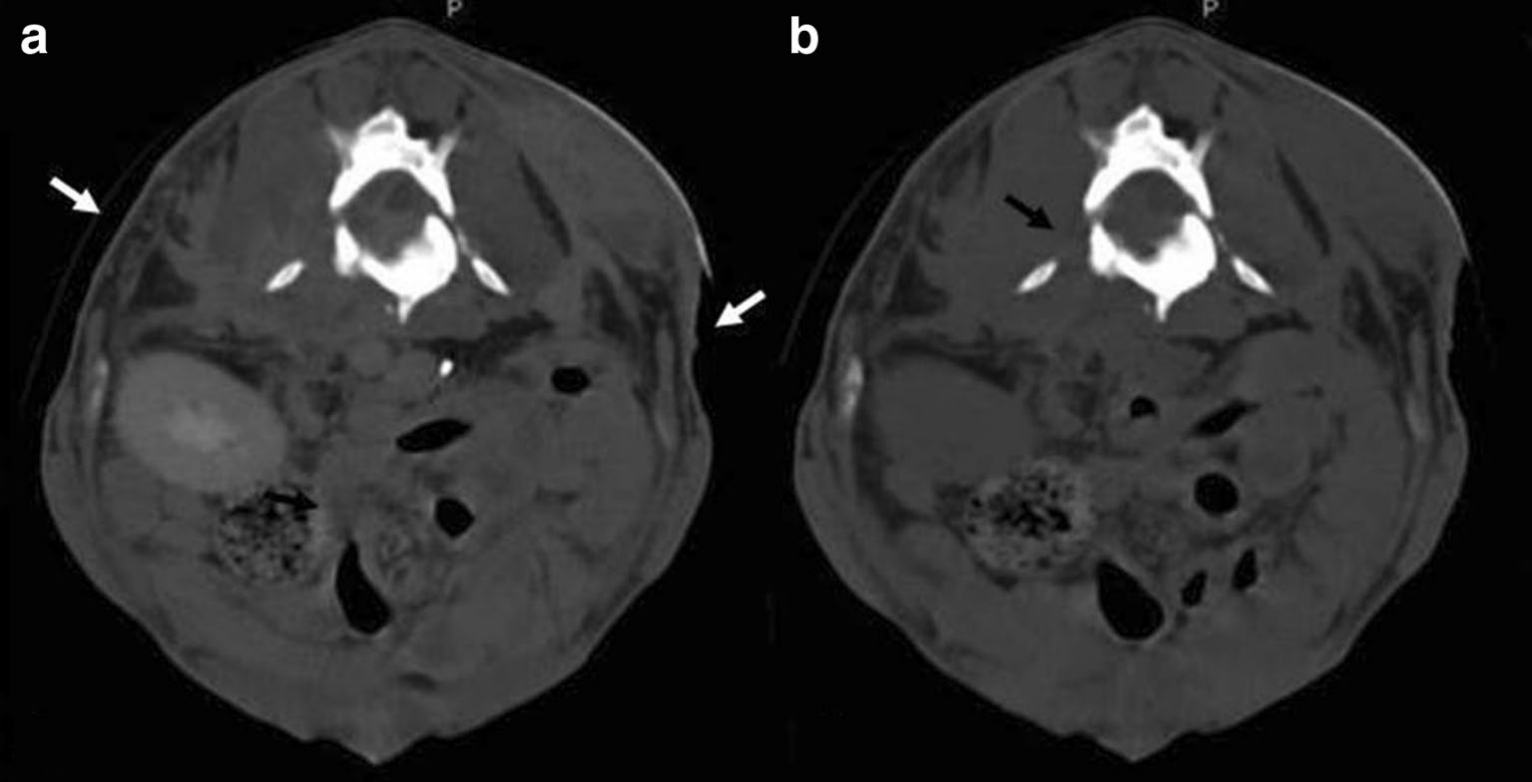
Intravenous contrast-enhanced (**a**) and non-enhanced (**b**) transverse CT projections. The images are oriented with dorsal at the* top* and the animal’s right to the left of the image. Both sections are at the level of L3–L4, and show irregularity and inhomogeneity of the iliopsoas muscles, with severe osteolysis of the vertebral body of the lumbar vertebra (L3) involving the spinal canal (*black arrow*). There is also discontinuity of the soft tissue of both the flanks indicating bilateral subcutaneous fistulae (*white arrows*)

Two dogs with clinical signs compatible with hind limb paraparesis underwent radiographic and CT studies of the thoracic and lumbar spine before the surgery. Radiographic abnormalities included signs of discospondylitis and osteomyelitis (Fig. 7). On CT, one of these dogs showed mild to moderate irregular bone proliferation in the ventral periosteum of L1–L3 lumbar vertebrae and the other showed severe ventral spondylosis with lysis and sclerosis of L3 and L4 end plates and vertebral bodies, extensive remodelling and partial collapse of the disc space, osteolytic and osteoproliferative changes including intense periosteal reaction of the vertebral body (Fig. 8). Both dogs showed inhomogeneity of the iliopsoas muscles. Computed tomography did not permit visualization of the plant material but only changes within the surrounding tissue associated with the inflammatory response. Neurological signs in both dogs were considered secondary to the foreign bodies and their impact on the lumbar vertebrae.

In all dogs, the plant material was successfully removed via ventral midline laparotomy in which intraoperative ultrasonography was used to direct the tips of the grasping forceps to the plant material and guide its removal. In 21/22 dogs, the plant material was entirely removed, whereas in one dog occurred fragmentation of the foreign body during the attempt of removing. A fragment was visualized by intraoperative ultrasonography of affected region and removed during the same surgery. In one dog, the grass awn was intraoperatively visualized more cranially than it had been during preoperative imaging. In 2 dogs with vertebral lesions, the plant material was found straddling the midline of the iliopsoas muscles adjacent to the affected vertebrae. Nevertheless, intraoperative ultrasonographic guidance resulted in successful removal of the migrating plant material. No dog had any intra- or postoperative complications.

All dogs were discharged from the hospital 3–5 days after the surgery with a prescription of antibiotics (cefadroxil 20 mg/kg PO q24 h, enrofloxacin 5 mg/kg PO q24 h, clindamycin 11 mg/kg PO q24 h, prescribed on the basis of microbial culture and susceptibly testing) for 10 days. In 2 dogs, the hospitalization lasted for 2 weeks because vertebral spondylosis and osteolysis secondary to the abscess required physical therapy and prolonged parenteral antibiotic treatment. Bacteriological isolation from the abscess was performed in all dogs. Microorganisms were isolated from abscesses of 12 dogs and included *Streptococcus* spp., *Staphylococcus aureus*, *Pseudomonas* spp., *Escherichia coli,* and *Proteus mirabilis*. None of the isolated microorganisms showed antibiotic resistance to the panel of antibiotics evaluated, including those isolated from patients receiving long-term antibiotics.

At a 1 month re-evaluation after discharge, the affected iliopsoas muscle in all dogs showed a progressive improvement of the ultrasonographic appearance (homogeneous parenchyma with typical fascicular architecture).

All 22 dogs had complete resolution of the clinical signs and resumed normal activity within 4–5 weeks after successful surgical removal of the plant material. Both dogs with neurological signs and osteomyelitis/discospondylitis recovered completely.

## Discussion

Our study provides evidence of the utility of intraoperative ultrasonography for removing plant material from iliopsoas muscles via ventral midline laparotomy. In previous reports of dogs with iliopsoas myositis, migrating plant material was often suspected or visualized by preoperative imaging, but was retrieved during surgical exploration in only some cases. The cause of this retrieval failure can only be speculated upon: it is possible that in these cases, the plant material had migrated from the abscess and exited via the fistula prior to surgical exploration, or that the plant material was not located within the abscess but elsewhere in the musculature or lumbar parenchyma during surgical exploration. In either situation, intraoperative ultrasonography would have likely guided the surgeons to the plant material or conclusively demonstrated its absence, as we have demonstrated in this study, increasing the rate of successful retrieval of plant material. When a grass awn was ultrasonographically identified, intraoperative ultrasound allowed the surgeon to grasp the tip of the grass awn after making a precise incision through the muscle, minimizing the risk of fragmentation and iatrogenic muscle damage. To the author’s knowledge, this is the first report of such a surgical strategy for removing plant material from iliopsoas muscles.

Oronasal ingestion or inhalation of the grass awns, especially in hunting dogs (which represented 100% of our cohort), commonly causes respiratory disease during spring and summer [13, 24, 25]. In our cases, respiratory disease signs and fever were recorded in 12/22 dogs during spring or summer season: these historical findings could be useful for the clinician approaching dogs with this clinical condition. Acute inhalation can go unnoticed by the owner, resulting in subsequent migration through the airways into the lung and then into the pleural space, pericardium, retroperitoneal cavity, iliopsoas muscles, or out through the thoracic/abdominal wall [15, 20, 21, 25–29]. This unidirectional migratory characteristic of grass awns is attributable to their backward-pointing barbs and fusiform shape [15, 20, 21, 25–29]. Grass awns cause severe and septic tissue reactions and variable clinical signs, depending upon their location. Grass awn migration into the iliopsoas muscles commonly causes local inflammation. Grass awns introduce bacteria, incite a foreign body response, interfere with local host defences and provide a nidus for chronic, infections [13]. Similar to previous reports [11, 13, 18], the most relevant clinical signs were flank swelling and pain, pyrexia and depression: these clinical signs are the consequence of septic tissue reaction secondary to the plant material migration, but are not specific for foreign-body-related iliopsoas myositis.

Ultrasonography is a safe, readily available, and non-invasive diagnostic technique that can be used to identify anatomic landmarks for planning and guiding a surgical approach for removing plant material [14, 15, 17, 20–22]. In contrast to CT or MRI, abdominal ultrasonography can be performed without anaesthesia. Moreover, CT and MRI are less frequently available to clinicians and require more advanced training for their use. Ultrasonographic findings in sublumbar migration are characterized by an enlargement of the affected iliopsoas muscles (when compared to the contralateral muscle), and an unstructured hypoechoic appearance with anechoic areas of variable size and number. Hyperechoic structures, typically as spindle-shaped casting an acoustic shadow, are frequently surrounded by this anechoic area and are consistent with a grass awn [13]. Similar to previous reports, identification of the plant material in dogs of the present study was enhanced by a surrounding hypoechoic region of fluid associated with an inflammatory response [15, 20–22].

Studies using CT examinations to characterize iliopsoas abscesses were able to detect plant material in only 38–47% of dogs in which plant material was found at surgery [16, 18]. This low rate of detection was attributed at least in part to local inflammation preventing visualization of the foreign body [18]. Holloway et al. reported hypointense muscle lesions consistent with foreign material on MRI in five patients but foreign material was ultimately identified in only two of these patients [8]. The authors concluded that the specificity of MRI in identifying small foreign objects appears to be low.

In 11 dogs in our study, the plant material was not removed during the initial surgery performed by the referring veterinarians. These dogs had undergone surgical exploration via lateral or ventral midline laparotomy without pre- or intraoperative ultrasonography. This suggests that our approach, utilizing both preoperative and intraoperative ultrasonographic localization and guidance, increases the likelihood of successful plant material removal. In all dogs, the plant material visualized pre- and intraoperatively was removed. Preoperative ultrasonography allowed us to identify the cause of the iliopsoas myositis and provided valuable landmarks for surgical approach. In one dog, immediate, intraoperative, post-removal scanning identified a remnant that was quickly removed before closure—had this not been performed, there is a high probability that clinical signs would have persisted in this dog. Therefore, it can be speculated that intraoperative ultrasonography is important to not only guide the surgical approach, but to confirm complete removal of the foreign body, especially where fragmentation of the plant material is suspected.

Some authors suggest that surgical exploration and debridement of the affected region, after a preoperative CT scan, can successfully resolve the problem, even if the plant material is not identified or removed [11, 18, 19]. In 2 cases we performed CT imaging to better investigate the neurological signs and associated lumbar vertebral abnormalities. In neither case was the foreign body visualized with CT imaging, consistent with findings of previous studies. We found that placing the probe directly on the affected muscle via the laparotomy allowed high-resolution visualization of the plant material without interference from the surrounding tissues (skin, fat, bone etc.). Therefore, we would suggest that even in dogs undergoing surgical exploration for iliopsoas myositis, suspected to be secondary to plant material, on the basis of CT or MRI findings, in which the foreign body is not visualized preoperatively, clinicians should perform intraoperative ultrasonography directly over the affected muscle to increase the probability of detecting plant material. Furthermore, this technique permits a more precise and less traumatic surgical approach to the plant material, and, in the case of grass awns, allows withdrawal of the grass awn in a manner that reduces risk of barb fragmentation.

The variations in grass awn length identified by ultrasonography and confirmed by surgical removal likely reflect the different species of grass awns present in Italy [15, 20, 21, 25]. However, similar species of grasses exist elsewhere, suggesting that our ultrasonographic descriptions are likely to be applied to grass awns in various regions of the world. The authors speculate the unusual plant material consistent with bramble twig behaved similarly to grass awns during its migration through lung to retroperitoneal cavity: thorns on the twig mimicked backward-pointing barbs of grass awns. Both bramble twig and fragments of grass awn, different ultrasonographically from typical spindle-shaped grass awns, were successful identified pre- and intraoperative ultrasonography. Ultrasonographic characteristics of this unusual plant material were useful in planning its surgical removal.

Aortic rupture has been described as a complication of surgical exploration of iliopsoas abscesses [11]. In the present study, intraoperative ultrasonography was useful to guide successfully plant material removal, but also to avoid possible damage of surrounded tissues by real-time monitoring of the surgical instruments (spinal needle, scalpel blade and forceps) as they were introduced into the muscle.

In dogs in the present study, a surgical approach by ventral midline laparotomy permitted excellent exposure and visualization of the affected area, closeness and proximity of the grass awn to the dorsal peritoneum and consequently to the surgeon’s hands, cleanliness of the surgical field and an accurate and targeted approach to preserve the iliopsoas muscles. These advantages cannot be always obtained by a lateral, transcutaneous approach to the sublumbar region. Moreover, an ultrasonographic probe can be easily positioned close to the diseased region with fewer imaging artifacts, compared to the lateral approach. In our study, no dogs suffered post-operative complications (e.g. peritonitis or wound dehiscence), and in all patients the clinical signs due to migrating plant material resolved. The Authors believe that an appropriate preparation of the surgical field by using wet laparotomy gauzes, and lavage and suction of the area around the grass awn reduces the incidence of complications. Our cases did not need extensive debridement during the surgical procedure or the establishment of drainage in the postoperative period with drainage tubes, because a limited amount of the fluid was recovered from the affected region. This might have been due, in part, to the long-term antibiotic therapy that most of these dogs had been subjected to before presentation to our institution. However, debridement or the establishment of drainage can be considered when iliopsoas myositis is markedly abscessated.

Our study had several limitations. The study was retrospective, with the limitations inherent in such study designs, although retrospective studies often provide the most suitable means of collecting sufficient data for evaluation of infrequently diagnosed disorders in a timely manner. Also, ultrasonography is a highly operator-dependent technique, and our results might not be readily extrapolated to similar situations where the methods involve other, less experienced, clinicians or different ultrasonography imaging systems or other imaging techniques. However, given the characteristic findings of the plant material we observed, we believe that most clinicians should be able to successfully image the iliopsoas muscles to locate migrating plant material. Finally, the study population was also somewhat small.

## Conclusions

Intraoperative ultrasonography is a safe and readily available tool that improves success of surgical removal of plant material within the iliopsoas muscles region via ventral midline laparotomy.

## Abbreviations

CT: computed tomography
MRI: magnetic resonance imaging

## Footnotes

1 MyLab 30 Vet Gold, Esaote, Genova, Italy.

2 Delta Med Medical Devices, Viadana, Italy.
